# Change lifestyle modification plan/transtheoretical model in non-alcoholic simple fatty liver disease: a pilot randomized study

**DOI:** 10.1186/s12876-022-02506-4

**Published:** 2022-11-23

**Authors:** Lijuan Li, Kun Hou, Mengya Yuan, Yan Zhang, Yang Zhang

**Affiliations:** 1 Department of Nursing, Zhangjiagang TCM Hospital, Nanjing University of Chinese Medicine, 215600 Zhangjiagang, China; 2 Department of Respiratory, Zhangjiagang TCM Hospital, Nanjing University of Chinese Medicine, 215600 Zhangjiagang, China; 3 Department of Gastroenterology, Zhangjiagang TCM Hospital, Nanjing University of Chinese Medicine, 215600 Zhangjiagang, China; 4 Department of Infectious Diseases, Zhangjiagang TCM Hospital, Nanjing University of Chinese Medicine, 215600 Zhangjiagang, China

**Keywords:** Transtheoretical model of behavior change, Non-alcoholic fatty liver disease, Lifestyle risk reduction, Health belief model, Self-management

## Abstract

**Background:**

Non-alcoholic simple fatty liver disease patients have very low compliance with almost all types of physical activities. A transtheoretical model-oriented lifestyle modification plan awakens the patient’s consciousness in the pre-intention stage. Aim to evaluate whether a management by stages of change plan based on the Transtheoretical Model and Stages of Change promoted behavior change for patients with non-alcoholic simple fatty liver disease.

**Methods:**

Patients with simple fatty liver diagnosed from July to December 2019 were randomly divided into the transtheoretical model and non-transtheoretical model groups. Primary outcome was change in health belief and health behavior based on questionnaires. Secondary outcomes included changes in blood lipids, body mass indexes, and waist circumference 12-months after intervention.

**Results:**

Of 200 enrolled patients 194 were analyzed (non-transtheoretical model group n = 98, transtheoretical model group n = 96). After intervention, total health belief scores (120.91 ± 4.94 vs. 118.82 ± 5.48) and total health behavior scores (131.71 ± 5.87 vs. 119.96 ± 7.12) were higher in the transtheoretical model group (all *P* < 0.05). Blood lipids, body mass index, and waist circumference more obviously improved in the transtheoretical model group (all *P* < 0.05).

**Conclusion:**

A transtheoretical model-based lifestyle modification intervention can be effectively applied to patients with non-alcoholic simple fatty liver.

**Clinical Research Registration Number:**

ChiCTR2100049354. The registration date is August 1, 2021.

**Supplementary Information:**

The online version contains supplementary material available at 10.1186/s12876-022-02506-4.

## Background

Nonalcoholic fatty liver disease (NAFLD) is characterized by excessive hepatic fat accumulation in the absence of any secondary causes including excessive alcohol consumption, use of steatogenic medication, viral infection, or hereditary disorders [1]. NAFLD is progressive and has a wide spectrum of histological characteristics from steatosis to nonalcoholic steatohepatitis (NASH), characterized by inflammation and fibrosis [2]. Once NASH has developed, progressive fibrosis may lead to cirrhosis and hepatocellular carcinoma [2]. Non-alcoholic simple fatty liver disease is the mildest disease state of hepatocyte steatosis in the course of pathological development of NAFLD [3]. Once simple fatty liver disease develops to the stage of fatty liver hepatitis, the related death rate could reach 11.77 cases in every 1,000 people each year [4]. At the simple fatty liver disease stage, appropriate nursing interventions, combined with diet and exercise instruction, psychological care, and health education are of great significance to the progression and treatment of NAFLD [5]. Such early intervention in the simple fatty liver disease stage is essential to prevent its further development into fatty liver hepatitis, cirrhosis and even liver cancer. However, most patients and some clinicians do not have sufficient awareness of the dangers of the disease [6].

Epidemiological surveys have shown that NAFLD patients have very low compliance with almost all types of physical activities recommended by the guidelines [7]. Therefore, it is difficult to promote and maintain patients’ behavior changes. The Transtheoretical Model and Stages of Change was proposed by the American psychologist Prochaska. It focuses on the subject’s psychological needs and the process of behavior change, and believes that behavior change is a phased, spiral, and gradual development process [8]. Individualized interventions are given according to the patient’s behavior transition stage to ensure that the patient’s self-management ability is spirally increased and maintained [9]. A transtheoretical model-oriented lifestyle modification plan awakens the patient’s consciousness in the pre-intention stage, enables the patient to have a clear understanding of their own disease, thereby generating emotions for positive changes. The core of its behavioral intervention is the change of philosophy, which is consistent with the processes of “concept-behavior” and “passive-active”, thereby enhancing patients’ health beliefs [10].

The aim of this study was to investigate the efficacy of management on a lifestyle modification plan for patients with simple fatty liver disease based on the transtheoretical model promoted behavior change and related physiological and biochemical indicators during 12 months follow-up.

## Methods

### Study design and subjects

This was an exploratory clinical trial on simple fatty liver disease patients diagnosed at the Health Administration Center of a tertiary hospital in Zhangjiagang City from July to December 2019. All simple fatty liver disease patients diagnosed in the study period were enrolled. This study was approved by the Hospital of Nanjing University of Traditional Chinese Medicine, with the serial number of 2020-5-2. All patients signed the informed consent.

The inclusion criteria were as follows: (1) Aged between 18 and 60; (2) Patients diagnosed with simple fatty liver disease according to ultrasound imaging (3) Basic ability in reading, writing and understanding. The exclusion criteria were: (1) Women during pregnancy or breastfeeding; (2) Patients with anxiety, depression and mental illness history; (3) Patients with motor dysfunction; (4) Patients who were taking liver-protecting drugs and lipid-lowering drugs; (5) Liver function was normal or basically normal, that is, alanine aminotransferase (ALT), aspartate aminotransferase [11], γ-glutamyl transpeptidase (GGT) did not exceed the normal range of 10u/L.

Generally, the diagnostic criteria for NAFLD are most accurately assessed by liver biopsy for evidence of the degree of liver steatosis, liver cell damage, inflammatory necrosis and fibrosis. However, because liver puncture is an invasive operation, with some adverse events, it is not universally recommended for patients with suspected steatosis. Therefore, this study applied ultrasound imaging standards as the diagnostic basis of simple fatty liver disease if item (a) was met and at the same time anyone or two of item (b) or item (c) was met as follows: (a) The echo from the front field of the liver was enhanced, showing a “bright liver”, which was stronger than the echoes from the spleen and the kidney; (b) The echo from the distant field of the liver was weakened; (c) The structure of the intrahepatic biliary tract was unclear [11]. In addition, the following definitions of “non-alcoholic” and “non-alcoholic fatty liver disease” were combined: (1) In the past 12 months, the amount of ethyl alcohol consumed by the patient was less than 210 g per week for men and less than 140 g for women; (2) Diseases such as viral hepatitis, autoimmune hepatitis, hepatolenticular degeneration, congenital lipid atrophy, hypothyroidism, celiac disease and other specific diseases that can lead to fatty liver were excluded; (3) The patients were not taking medicines such as amiodarone, methotrexate, sodium valproate, glucocorticoids; (4) Bullous or bullous dominating steatosis had affected more than 5% of hepatic cells, with or without mild non-specific inflammation [12].

### Interventions

The patients were randomized into two groups by the patient’s physical examination numbers through a random number table method. The transtheoretical model group received lifestyle modification based on Transtheoretical Model and Stages of Change, and the control group received routine health guidance and follow-up intervention (described below). To ensure the uniformity of the analysis of the 2 groups of patients, before the start of the study, 3 members of the research team were trained in relation to the unification of the instructions for filling in the scale, the instruction language, and the evaluation methods, and whether the investigators had concealed the grouping of patients. The investigator (without knowing the behavior change stage of the patient) explained the survey questions to the patient one by one, asked the patient to fill it out and retrieve it on the spot. If the patient could not fill it out, the investigator would ask for the patient’s permission and filled it out on their behalf and give them feedback.

For the patients in the transtheoretical model group, a transtheoretical model-oriented lifestyle amendment plan was formulated. The transtheoretical Model and Stages of Change believes that individual behavior changes can be divided into 5 stages [13]: (1) Pre-intention stage: The patient has poor knowledge of healthy lifestyles, is not aware of the harm caused by the unhealthy lifestyles, and has no plan to change within 6 months; (2) Intention stage: The patient has realized the shortcomings in his/her daily lifestyle and plans to change it within 6 months; (3) Preparation stage: The patient has established a goal for changing his/her lifestyle, and occasionally controls diet and strengthens exercise, which has not been regular; (4) Action stage: The patient has been able to observe a healthy lifestyle, but this has been no more than 6 months; (5) Maintaining phase: The patient has gotten through behavioral changes, which means they are strictly abiding by the principles of diet and exercise for more than 6 months. Ten intervention strategies can be implemented corresponding to these 5 stages. Pre-intention stage: (1) Awareness awakening, (2) Vivid relief, (3) Self-efficacy; Intention stage: (4) Self-reassessment, (5) Environmental reassessment; Preparation stage: (6) Self-liberation, (7) Helping relationships; Action stage: (8) Counter-condition; Maintaining phase: (9) Strengthen management, (10) Stimulus control. For these 10 intervention strategies, there are a series of matching intervention measures, which can be modified according to the actual situation during the implementation process. See Supplementary Table 1.

The patient’s behavioral transition stage was assessed in combination with the different performances at each stage of the Transtheoretical Model and Stages of Change. A behavioral change assessment questionnaire was used for evaluation [14], which was developed by the Cancer Prevention and Research Center of the United States and translated into Chinese by Guo Zhiping et al. In order to ensure the uniformity of the survey of the two groups of patients’ scales, the instructions, guiding terms, and evaluation methods were filled in uniformly before the survey. The investigators distributed them to the patients on the spot and explained them one by one. The patients filled them out and returned them directly. If the patients could not fill them out, the investigators consulted the patients, and their relatives filled them out, and checked them back with the patients for confirmation. The content of the questionnaire was “Please tell us your actual status truthfully according to the options”. Before starting the intervention, the definition of each stage was explained to the patient. The investigator evaluated it accordingly and divided the patient into the pre-intent stage group, the intention stage group, the preparation stage group, the action stage group, and the maintaining stage group according to the behavior change stage they are in. During the first 6 months of the study, the evaluation was conducted once every month, and once every 2 months for the next 6 months. Patients were graded as good behavioral changes if transformation of thought and behavior met the criteria for entering the next stage; medium behavioral change if after the intervention, thought and behavior changed only a little and stay at the original stage; and bad behavioral changes if the patient’s behavior regressed instead of being paid attention to. For patients with good behavioral changes, encouragement and affirmation were given, and they proceeded to the next stage in order. For patients who had remained in a stage over two evaluations, the reasons for not progressing were analyzed and interventions were performed again until the patient reached the maintaining stage. Evaluation and grouping ran throughout the entire research stage.

Different intervention measures were implemented for patients at different stages, including WeChat groups, lectures, model demonstration, consultation with an expert, group discussions, peer education, telephone follow-ups, and introductory visits. The research team consisted of 1 expert in gastroenterology, 1 expert in sports medicine, and 1 expert in nutrition, and 5 experts in nursing. All members of the team received training on Transtheoretical Model and Stages of Change related content and implementation methods through lectures, seminars and out-of-office learning.

For patients in the control group, routine health guidance and follow-up intervention were adopted. After the physical examination results were issued, materials relating to healthy lifestyle publicity for fatty liver disease were distributed to the patients, lectures for health education were held, and WeChat groups were organized to give guidance and consultations on disease knowledge, diet types, exercise plans, and emotional psychology. The patients were advised to quit smoking and limit alcohol, maintain a good life schedule and mood. Follow-ups by phone were performed for 12 months, once each month for the first 6 months and once every 2 months in the last 6 months, which were mainly intended to follow the patients’ lifestyles -including their smoking and alcohol habits- and give instructions, and provide help when patients met any difficulties.

### Baseline collection

Baseline information of all the patients included gender, age, degree of education, profession, residence, concomitant symptoms of diabetes, impaired fasting glucose/impaired glucose tolerance, and high blood pressure, liver function indexes, and imaging diagnosis were collected after patients’ enrollment.

The diagnostic criteria of diabetes were having typical symptoms of diabetes, plus fasting blood glucose ≥ 7.0mmol/l, or random blood glucose ≥ 11.1mmol/l, or oral glucose tolerance test (OGTT) 2 h blood glucose ≥ 11.1mmol/l, or HbA1c ≥ 6.5%. The diagnostic criteria for impaired fasting blood glucose were fasting blood glucose 6.1 ~ < 7.0mmol/l, OGTT 2 h blood glucose < 7.8mmol/l. Diagnostic criteria of abnormal glucose tolerance: fasting blood glucose < 7.0mmol/l, OGTT 2 h blood glucose 7.8 ~ < 11.1mmol/l. High blood pressure was defined as systolic blood pressure ≥ 140mmHg, diastolic blood pressure ≥ 90mmhg.

## Outcomes

The primary outcome of the study was the change in both health belief and health behavior between the two groups at 12 months after intervention. The secondary outcomes of the study were changes in biochemical and physical indicators at 12 months after intervention. These included blood lipids, body mass index (BMI), and waist circumference.

For assessment of health belief, Ji Shaoyan’s [15] Cross-culturally Adapted New Version Health Belief Scale was used to score patients. The scale has 48 items in 5 dimensions, of which items 1–10 are personal health beliefs, and 11–17 are senses of health implementation ability, 18–23 senses of control, 24–37 are sense of having resources, and 38–48 are senses of being threatened. The Likert 5-level scoring method was used, with a total score of 48 to 240. The Cronbach’s α coefficient of the scale is 0.935.

For assessment of health behavior, the Health Promoting Lifestyle Scale II (HPLP-II) [16] was used to score the patients. HPLP-II is mainly used to evaluate the health behavior level of a population, including maintaining and promoting healthy behaviors. The scale includes 52 items in 6 dimensions: self-realization, health responsibility, diet, exercise, interpersonal support, and stress management. Using the Likert 4-level scoring method, the scale is 1 to 4 points from “Never” to “Always”, with a total score of 52 to 208. The higher the score, the better the healthy behavior. The level of healthy behavior is divided into excellent (172 points to 208 points), good (132 points to 171 points), average (92 points to 131 points), and poor (52 points to 91 points) based on the total score of the scale. The levels of healthy behavior of the 2 groups of patients before intervention and after 12 months of intervention were compared.

Four items of blood lipids were measured and collected at pre-intervention or post intervention at 12 months. The patient had a light diet the day before and fasted for more than 8 h before the venous blood was drawn. The blood was tested within 2 h using the same biochemical analyzer. According to the stratification standard of Chinese adult blood lipid level [17], the appropriate ranges are serum total cholesterol (TC) < 5.18mmol/L, triacylglycerol (TG) < 1.76mmol/L, high-density lipoprotein (HDL-C) ≥ 1.04 mmol/L, low-density lipoprotein (LDL-C) < 3.37mmol/L. A liver ultrasound was conducted on the same day as blood lipid measurement. A standardized method was used to measure the patient’s BMI and waist circumference. The BMI equals to actual body mass (Kg)/height^2^ (m^2^), and the waist circumference measurement takes the abdominal circumference at the midpoint of the connection between the lower edge of the rib and the iliac ridge. Weight and height and waist circumference values are accurate to two digits after the decimal point. The measured values of body mass index and waist circumference of patients at pre-intervention or post intervention at 12 months were compared.

### Statistical analysis

SPSS 21.0 (IBM, Armonk, NY, USA) was used for statistical analysis. For quantitative data with a normal distribution, means and standard deviations (X±s) were used for description; the independent t-test was used for comparisons between the two groups. Two-way repeated analysis of variance (ANOVA) was used to find differences between groups and the effects of time and Time×Group interactions after 12 month on simple fatty liver disease patients. After this, ANOVA models were constructed to determine the effects of group allocation and change in simple fatty liver disease patients. All statistical analyses were two-sided. P-values < 0.05 were considered statistically significant.

## Results

### Baseline characteristics

The study included 200 cases at enrollment, randomly assigned to 100 cases in the control group and 100 cases in the transtheoretical model group. During the study, two patients in the control group and four in the transtheoretical model group dropped out of the study analysis due to poor compliance and voluntary interruption of follow-up. Therefore, a total of 194 patients finally completed the study and were included in the analysis. Among them, there were 166 male patients and 28 female patients, aged 24 to 60 years old, with a mean age of 42.46 ± 8.64 years. There were 98 cases in the control and 96 cases in the transtheoretical model group. There was no statistical significance in the difference between the 2 groups of patients in any of the baseline characteristics including gender, age, education level, and occupation (*P* > 0.05), in addition the two groups showed similar numbers of cases with diabetes, impaired fasting glucose/impaired glucose tolerance, and high blood pressure (*P* > 0.05). Liver function tests and imaging diagnoses of simple fatty liver disease were also similar (*P* > 0.05) as shown in Table 1.

**Table 1:** Demographic and clinical characteristics of patients in the two groups

Clinical characteristics	TMM, n = 96	Non-TMM, n = 98	***t/χ*** ^**2**^	***P***
Gender [*n* (%)]				
Male	84(87.50)	82(83.67)	0.575	0.448
Female	12(12.50)	16(16.33)		
Age (year, mean ± standard deviation)	42.03 ± 8.53	42.88 ± 8.77	0.681	0.497
Degree of education [*n* (%)]				
Junior college and above	53(55.21)	56(57.14)	0.074	0.964
Middle school and high school	41(42.71)	40(40.82)		
Primary school and below	2(2.08)	2(2.04)		
Profession[*n* (11)]				
Staff members of government organs and public institutions	19(19.79)	18(18.37)	0.092	0.955
Management personnel of enterprises and public institutions	26(27.08)	26(26.53)		
Enterprise staffs	51(53.13)	54(55.10)		
Residence[*n* (11)]				
Cities and towns	67(69.79)	68(69.39)	0.004	0.951
Villages	29(30.21)	30(30.61)		
Diabetes	2(2.08)	3(3.06)	0.185	0.667
Impaired fasting glucose/ impaired glucose tolerance	1(1.04)	2(2.04)	0.318	0.573
High blood pressure	3(2.13)	2(2.04)	0.227	0.634
Liver function index				
ALT(u/L)	37.50 ± 20.67	36.95 ± 22.69	-0.177	0.860
γ-glutamyl transpeptidase (u/L)	53.31 ± 37.86	54.26 ± 44.07	0.160	0.873
AST(u/L)	25.17 ± 8.32	25.59 ± 9.69	0.974	0.744
Imaging diagnosis basis [*n* (%)]				
(1)+(2)	36 (37.50)	39 (39.80)	0.572	0.751
(1)+(3)	46 (47.92)	42 (42.86)		
(1)+(2)+(3)	14 (14.58)	17 (17.34)		

ALT: alanine aminotransferase; AST: aspartate aminotransferase; TMM: Transtheoretical Mode

High blood pressure was defined as systolic blood pressure ≥ 140mmHg, diastolic blood pressure ≥ 90mmhg

Imaging diagnosis basis: (1) The echo from the front field of the liver was enhanced, showing a “bright liver”, which was stronger than the echoes from the spleen and the kidney; (2) The echo from the distant field of the liver was weakened; (3) The structure of the intrahepatic biliary tract was unclear

### Primary outcomes

Both groups showed a significant change in health belief over the 12 months before (T1) and after intervention (T2). The scores for personal health belief, sense of having implementing ability, sense of control, sense of having resources, sense of being threatened, and total score were all higher after intervention in both groups (all *P* < 0.001). Comparison between groups showed that scores for personal health belief, sense of having implementing ability, sense of being threatened, and total score were higher in the transtheoretical model group (all *P* < 0.001) but sense of control, sense of having resources were not significantly different between the groups. There was a significant interaction between time and group for each of the scores for health belief (all *P* < 0.05) (Table 2).

**Table 2:** Pre- (T1) and post-assessment (T2) scores on primary outcome measures and transtheoretical model and control effectiveness

	TTM, n = 96			Non-TTM, n = 98				***Time***		***Group***		***Interaction between*** ***time and group***	
	T1, Mean ± SD	T2, Mean ± SD	Cohen’s d	T1, Mean ± SD	T2, Mean ± SD		Cohen’s d						
Scores of health belief													
Personal health belief	27.11 ± 1.63	27.89 ± 1.26	0.535	26.88 ± 1.74	27.36 ± 1.68		0.281		F = 128.80 P < 0.001		F = 18.83 **P < 0.001**		F = 7.11 P = 0.009
Sense of having implementing ability	17.48 ± 1.67	17.94 ± 1.42	0.297	17.20 ± 1.55	17.43 ± 1.44		0.154		F = 78.91 P < 0.001		F = 25.82 **P < 0.001**		F = 10.20 P = 0.002
Sense of control	17.18 ± 1.45	17.71 ± 1.24	0.393	17.23 ± 1.53	17.51 ± 1.34		0.195		F = 35.94 P < 0.001		F = 3.62 P = 0.06		F = 11.35 P = 0.001
Sense of having resources	32.55 ± 1.61	33.15 ± 1.47	0.389	32.78 ± 1.72	33.09 ± 1.55		0.189		F = 60.51 P < 0.001		F = 0.59 P = 0.443		F = 11.11 P = 0.001
Sense of being threatened	23.48 ± 2.74	24.23 ± 2.42	0.290	23.07 ± 2.84	23.43 ± 2.60		0.132		F = 90.43 P < 0.001		F = 23.99 **P < 0.001**		F = 17.65 P < 0.001
Total score	117.80 ± 5.68	120.91 ± 4.94	0.735	117.16 ± 6.06	118.82 ± 5.48		0.287		F = 287.39 P < 0.001		F = 48.45 **P < 0.001**		F = 56.74 P < 0.001
Scores of health behavior													
Self-realization	22.02 ± 1.81	22.25 ± 1.77	0.128	21.80 ± 1.94	22.22 ± 1.89		0.219		F = 46.95 P < 0.001		F = 2.27 P = 0.135		F = 4.40 P = 0.039
Health responsibility	23.40 ± 2.88	25.83 ± 2.55	0.893	23.09 ± 2.40	23.65 ± 2.22		0.242		F = 132.39 P < 0.001		F = 39.15 **P < 0.001**		F = 64.32 P < 0.001
Exercise	15.40 ± 2.73	20.04 ± 3.07	1.597	15.09 ± 2.68	15.88 ± 2.90		0.283		F = 14.18 P < 0.001		F = 50.43 **P < 0.001**		F = 34.95 P < 0.001
Nutrition	17.27 ± 2.58	23.13 ± 1.85	2.610	17.69 ± 3.11	18.38 ± 3.35		0.213		F = 753.67 P < 0.001		F = 79.56 **P < 0.001**		F = 503.70 P < 0.001
Interpersonal relation	21.94 ± 1.81	22.44 ± 1.84	0.274	21.73 ± 1.89	22.27 ± 2.00		0.278		F = 38.01 P < 0.001		F = 5.39 **P = 0.022**		F = 0.004 P = 0.949
Stress management	17.04 ± 2.03	18.02 ± 2.26	0.456	17.13 ± 2.23	17.56 ± 2.12		0.198		F = 77.78 P < 0.001		F = 5.45 **P = 0.022**		F = 12.88 P = 0.001
Total score	117.06 ± 6.43	131.71 ± 5.87	2.380	116.54 ± 7.24	119.96 ± 7.12		0.476		F = 227.78 P < 0.001		F = 1001.60 **P < 0.001**		F = 378.44 P < 0.001

TTM: Transtheoretical Model

Time: postintervention compared with pre-intervention

Group: TTM group compared with control group

The results of the changes of health behavior scores before (T1) and 12 months after intervention (T2) demonstrate that self-realization, health responsibility, exercise, nutrition, interpersonal relation, stress management, and total score were all significantly improved with time (all *P* < 0.001). While scores for health responsibility, exercise, nutrition, interpersonal relation, stress management, and total score (all *P* < 0.05) were improved more in the observation than the control group. There was a significant interaction between time and group for each of the scores for health responsibility, exercise, nutrition, stress management, and total score (all *P* < 0.05) (Table 2). In addition, the health behavior level was improved after intervention in TTM group (P = 0.014) and TTM group had a better healthy behavior level than Non-TTM group after intervention (Supplementary Table 2).

### Secondary outcomes

Both groups showed decreased levels of TG (*P* < 0.001), TC (*P* < 0.001), and LDL-C (*P* < 0.001) but increased HDL-C (*P* = 0.008) from before to 12 months after intervention. The level of LDL-C was decreased more in the transtheoretical model group (*P* = 0.004). All of the lipid levels showed a significant interaction of time and group suggesting that the transtheoretical model intervention improved blood lipid measurements. In addition, the changes in BMI and waist circumference in males and females in the control and observation groups. Both these measures were significantly decreased 12 months after intervention compared to before intervention (all *P* < 0.05). However, waist circumference in males decreased more in the transtheoretical model group than the control group (*P* = 0.046). Both BMI and waist circumference measures showed a significant interaction between time and group, suggesting that the transtheoretical model helped reduce these measures (Table 3).

**Table 3:** Pre- (T1) and post-intervention (T2) on secondary outcome measures including blood lipids, body mass index (BMI), and waist circumference and transtheoretical model and control effectiveness

Secondary Outcomes		TTM group (n = 96)				Non-TTM group(n = 98)			**Time**	**Group**	**Interaction between time and group**
		T1	T2	Cohen’s d		T1	T2	Cohen’s d			
TG (mmol/L)		2.12 ± 1.43	1.66 ± 0.99	0.374		2.08 ± 1.14	1.96 ± 0.99	0.112	F = 85.97 P < 0.001	F = 0.52 P = 0.471	F = 25.38, P < 0.001
TC (mmol/L)		5.16 ± 0.91	4.61 ± 0.83	0.632		5.28 ± 1.18	5.00 ± 1.06	0.250	F = 144.47 P < 0.001	F = 3.30 P = 0.072	F = 18.65, P < 0.001
LDL-C (mmol/L)		3.56 ± 0.69	3.15 ± 0.59	0.639		3.78 ± 0.96	3.59 ± 0.93	0.201	F = 116.22 P < 0.001	F = 8.86 **P = 0.004**	F = 26.84, P < 0.001
HDL-C (mmol/L)		1.19 ± 0.27	1.30 ± 0.29	0.393		1.20 ± 0.25	1.21 ± 0.24	0.041	F = 7.38 P = 0.008	F = 0.98 P = 0.323	F = 6.64, P = 0.011
BMI (kg/m2)	Male	27.04 ± 2.89	25.18 ± 2.39	0.701		26.93 ± 3.15	26.37 ± 2.78	0.189	F = 94.69 P = 0.014	F = 6.37 P = 0.471	F = 45.12, P < 0.001
	Female	24.82 ± 3.47	21.89 ± 1.49	1.097		24.04 ± 2.21	23.40 ± 2.11	0.296	F = 33.48 P < 0.001	F = 0.03 P = 0.857	F = 14.54, P = 0.003
Waistline (cm)	Male	93.54 ± 6.44	87.28 ± 5.36	1.057		92.53 ± 6.51	90.68 ± 6.18	0.291	F = 244.32, P < 0.001	F = 4.12 P = **0.046**	F = 105.71, P < 0.001
	Female	83.14 ± 6.78	75.52 ± 4.87	1.291		82.23 ± 5.41	80.29 ± 6.55	0.323	F = 19.61 P = 0.001	F = 0.07 P = 0.803	F = 7.71, P = 0.018

Abbreviation: BMI, body mass index, TTM: Transtheoretical Model, TG: Triglyceride, TC: total cholesterol, LDL-C: low-density lipoprotein cholesterol, HDL-C: high-density lipoprotein cholesterol

Time: postintervention compared with pre-intervention

Group: TTM group compared with control group

## Discussion

This study showed that intervention with the Transtheoretical Model and Stages of Change increased personal health belief, sense of implementation capability, sense of being threatened, and total health belief scores more than routine health guidance. The transtheoretical model also increased the number of cases with excellent and good health behavior, and blood lipids, body mass index, and waist circumference improved. These findings suggest that the transtheoretical model improved the health beliefs, established healthy behaviors, and reduced the weight of patients with simple fatty liver disease.

Currently, establishment of a healthy lifestyle is the main intervention for NAFLD treatment. This has recently led to the innovative nursing models of NAFLD, such as behavioral intervention models, solution focused approaches, and problem-based learning health education models [18]. They have a certain effect on motivating patients to establish healthy behaviors. But these models have not been personalized for patients in terms of health beliefs, which often leads to a lack of confidence and compliance and limits the adherence duration. Only when a person has the motivation to change from deep in his heart, can he actually put it to action. The core of implementing a transtheoretical model-oriented lifestyle modification and intervention for simple fatty liver disease patients is to change the patient’s philosophy and enhance the patient’s decision-making ability for behavior change, thereby motivating patients to establish and maintain healthy lifestyles [19]. The results of the study showed that the level of health behavior after transtheoretical model was better than that of the controls who had routine health guidance.

This study also suggested that a transtheoretical model-oriented lifestyle modification plan can improve the physiological and biochemical indicators of simple fatty liver disease patients. Because NAFLD is a liver manifestation of obesity and metabolic syndrome, lowering BMI through lifestyle adjustments such as diet and exercise can interfere with disease progression [20]. Sun Guili [21] et al. used “5 + 2” intermittent fasting therapy to improve the severity of fatty liver in patients with non-alcoholic fatty liver. But most of the subjects found intaking only 1/4 of the energy of non-fasting days on fasting days difficult for a long period of time. In this study, the transtheoretical model-oriented lifestyle modification plan helped make patients fully aware of the difficulties that may be encountered in behavior change, encouraged family members to participate in the intervention process, and provided links to other external resources to support adherence [22]. The results of the study showed that the 4 tested values of blood lipids in the transtheoretical model group after intervention improved more than those in the controls, and the difference between the 2 groups was statistically significant. According to the weight criteria for Chinese adults, BMI ≥ 28 means obesity, 24.0 ≤ BMI < 28 means overweight, and 18.5 ≤ BMI < 24.0 means normal weight [23]. In this study there were significantly fewer obese and overweight patients after intervention in the transtheoretical model group than in the controls. According to the national essential public health service standards: women with waist circumference of ≥ 85 cm and men with waist circumference of ≥ 90 cm have central obesity (abdominal obesity) [24]. Significantly fewer patients had central obesity in this study after intervention with the transtheoretical model compared to the controls.

This study has some limitations. As a pilot study, the sample size was quite small so this may limit the power of the study in detecting differences between the two groups and may result in uneven sampling at the different stages of change. However, the results provide guidance towards the design of future study from multi-centers with larger sample size. The follow-up was only 12 months, a longer time period would be needed to establish long-term adherence to the changes in lifestyle. The evaluation of lipid metabolism-related indicators can be as accurate as the detection of visceral fat, but other indicators of liver function and liver steatosis would provide more information to promote the technology more effectively. Fibroscan® and liver biopsy (histopathology) results are needed to help evaluate the NAFLD progress.

## Conclusion

To sum up, the implementation of the transtheoretical model-oriented lifestyle modification and intervention for simple fatty liver disease patients can significantly improve their health belief and behavior, leading to improvements in blood lipids and decreased BMI and waist circumference. These health improvements are likely to prevent NAFLD progression or improve liver function by decreasing liver steatosis.

## Electronic supplementary material

Below is the link to the electronic supplementary material.

Supplementary Material 1. Supplementary Table 1. Transtheoretical Model and stages of change-oriented lifestyle intervention strategies and measures for simple fatty liver disease patients. Supplementary Table 2. Comparison of healthy behavior levels of the 2 groups (controls vs. transtheoretical model and stages of change) before and after intervention.

## Data Availability

The datasets generated and/or analysed during the current study are not publicly available due to their inclusion of personally identifiable patient information but are available from the corresponding author on reasonable request.

## List of abbreviations

NAFLD: Nonalcoholic fatty liver disease
NASH: nonalcoholic steatohepatitis
GGT: glutamyl transpeptidas
HPLP-II: Health Promoting Lifestyle Scale II
ANOVA: analysis of variance
