# Supplementary material for: Change lifestyle modification plan/transtheoretical model in non-alcoholic simple fatty liver disease: a pilot randomized study
Source: BMC Gastroenterol. 2022 Nov 23;22:483. doi: 10.1186/s12876-022-02506-4 (PMC9685906; doi:10.1186/s12876-022-02506-4)
Supplement: Supplementary file 1 — Supplementary Material 1. Supplementary Table 1. Transtheoretical Model and stages of change-oriented lifestyle intervention strategies and measures for simple fatty liver disease patients. Supplementary Table 2. Comparison of healthy behavior levels of the 2 groups (controls vs. transtheoretical model and stages of change) before and after intervention. [file 12876_2022_2506_MOESM1_ESM.docx]

**Supplementary Table 1.**

Transtheoretical Model and Stages of Change-oriented lifestyle intervention strategies and measures for simple fatty liver disease patients

| Stage of change | Intervention strategy for different stage | Corresponding intervention strategy |
| --- | --- | --- |
| Pre-intention stage | Awareness awakening - learn scientific concepts that can support healthy behaviors | Explain the pathological changes, disease development, and precipitating factors of non-alcoholic fatty liver degeneration, organize patients to visit the hospital, feel the pain of patients with liver cirrhosis or liver cancer, and help patients establish beliefs in healthy behaviors. |
|  | Vivid relief - experience the negative feelings brought about by unhealthy behaviors | Patients were asked to recall the physical and mental discomfort caused by bad lifestyles such as overeating, being drunk, staying up late, and the serious consequences of bad lifestyles on diseases and the benefits of healthy behaviors were emphasized. |
|  | Self-efficacy - building confidence with healthy behavior | Patients with good lifestyles and behaviors were invited to share their experience of successful changes, so that patients could understand that they can improve or recover their condition by establishing healthy behaviors, thereby generating self-efficacy. |
| Intention stage | Self-reassessment - understand the importance of establishing healthy behaviors | Patients were helped to analyze their own behavior, and they could tell the inconsistency of cognition and behavior, weigh the pros and cons of a bad lifestyle and a healthy lifestyle, and be guided to make the right decision. |
|  | Environmental reassessment - the distress caused by unhealthy behaviors to family and friends | Patients were guided to realize that if the condition develops further, it will bring huge medical expenses and troubles to family and friends due to the need for care. |
| Preparation stage | Self-liberation - make the decision to change in important occasions | Patients were helped to develop a behavior change plan. A behavior change agreement was signed with the patients, and patients were asked to promise on some important occasions to correct unhealthy behaviors, establish healthy behaviors, and liberate themselves. |
|  | Helping relationships - learn to use social support when changing unhealthy behaviors | WeChat groups were established. Patients who have questions or encounter difficulties in establishing healthy behaviors can consult experts in the group at any time. Patients were asked to memorize the telephone number of the researcher, and family members and friends were invited to participate in the supervision on patients, and at the same time inter-patients exchange meetings were organized to share successful experience and form a good helping relationship. |
| Action stage | Counter-condition - replace unhealthy behaviors with healthy behaviors | Detailed diet and exercise guidance plans and weight loss plans were formulated. Patients were encouraged to follow the recipe provided by the nutritionist to control their diet, and exercise according to the prescription provided by the sports medicine expert. At the same time, patients were requested to quit smoking and limit alcohol intake, reduce staying up late, maintain a good mood, and develop healthy behaviors. Counter-condition effect continues to take effect. |
| Maintaining stage | Strengthen management - continue to encourage changes in healthy behaviors | When patients can adhere to healthy behaviors, family support was sought and material or spiritual encouragement were given to the patients to strengthen behavior changes in the action stage, make patients feel the happiness of life brought by healthy behaviors, strengthen patients' confidence in maintaining and solidifying healthy behaviors. |
|  | Stimulus control - reduce or remove bad hints, increase positive hints | The patient’s family members were instructed that light dishes, mixing meat and vegetables, should be prepared at home, and when eating out, test reports and diet plans should be carried, so as to refuse alcohol and remind oneself to eat less greasy food. Researchers regularly conducted follow-ups by phone calls or home visits to stimulate the maintenance of their healthy behaviors. |

**Supplementary Table 2.** Comparison of healthy behavior levels of the 2 groups (controls vs. Transtheoretical Model and Stages of Change) before and after intervention

| Healthy behavior levels | TTM, N=96 | | Non-TTM, N=98 | |
| --- | --- | --- | --- | --- |
|  | T1, n(Duan et al.) | T2, n(Duan et al.) | T1, n(Duan et al.) | T2, n(Duan et al.) |
| Excellent | 4(4.17) | 9(9.38) | 5(5.10) | 6(6.12) |
| Good | 22(22.92) | 38(39.58) | 17(17.35) | 21(21.43) |
| Average | 54(56.25) | 41(42.71) | 58(59.18) | 56(57.14) |
| Poor | 16(16.67) | 8(8.33) | 18(18.37) | 15(15.31) |
| T1 vs T2 | **P=0.014**b | | P=0.845a | |
| Non-TTM vs TTM | | | | |
| T1 | P = 0.803c | | | |
| T2 | **P =** 0.019d | | | |

Note: Rank sum test for 2 groups (Non-TTM vs TTM) or paired rank sum test for before and after was used**.**

a: Comparison between Pre (T1) and post intervention(T2) in Non-TTM group;

b: Comparison between Pre (T1) and post intervention(T2) in TTM group.

c: Compared between the 2 groups (Non-TTM vs TTM) at Pre- intervention (T1);

d: Compared between the 2 groups (Non-TTM vs TTM) at Post- intervention (T2)
